# Toxicological considerations of nano-sized plastics

**DOI:** 10.3934/environsci.2019.5.367

**Published:** 2019-10-22

**Authors:** PA Stapleton

**Affiliations:** 1 Department of Pharmacology and Toxicology, Ernest Mario School of Pharmacy, Rutgers University, 160 Frelinghuysen Rd., Piscataway, NJ 08854, USA; 2 Environmental and Occupational Health Sciences Institute, 170 Frelinghuysen Rd., Piscataway, NJ 08854, USA

**Keywords:** toxicology, exposure, nanoplastics, microplastics

## Abstract

Undoubtedly, plastics have changed human existence. These pervasive products are used in nearly every field to include technological, biomedical, and domestic applications. Post-consumer plastic waste disposal leading to plastic pollution in landfills, waterways, and oceans represents a worldwide environmental challenge. Accumulation and continued material fragmentation from micro- to nanoplastics has identified concerns pertaining to environmental and human exposures and toxicity. While many studies have focused on particle fate and identification, the toxicological considerations must focus on the biological relevance of particle deposition within a particular organism, compartment, organ, and tissue. Further, concerns exist regarding the physical and chemical properties of the plastic particles during their production and/or degradation. In this mini-review we will discuss (1) particle characterization and assessment, (2) environmental concerns, and (3) human toxicity.

## 1. Introduction

Plastics are produced through the chemical and physical processing of naturally occurring constituents. Through polymerization and polycondensation, base constituents react together to form polymer chains, a process that can rarely be reversed. Therefore, once the reaction has occurred, these molecules cannot return to their previous basic form only be further processed or recycled to differing polymeric forms. Industrial chemicals may be added to the reaction to develop harder or more malleable results. Due to chemical stability, the environmental accumulation of plastics is on the rise and the research documenting these increases is receiving mainstream interest. Unfortunately, as identified in a recent editorial in Nature Nanotechnology, the laboratory and environmental toxicological assessments have not been completed, and overall, we simply do not know the outcomes [1].

## 2. Microplastic verses nanoplastic

The term “nanoplastic” is relatively novel. The first utilization of the term in a Web of Science search was within a 2004 abstract describing computational methods pertaining to material deformation [2]. As such, there has been some discussion in the literature regarding the definition of a ‘nanoplastic’. However, this is an important characterization for clarity as the field moves forward.

By definition, microplastics are plastic pieces that are less 5 millimeters (mm) in one dimension; therefore, nanoplastics would be considered ultrafine plastics that fall under this umbrella term. The discrepancy of terminology lies with how the nanoplastic produced. Nanoplastics in ecotoxicological settings are primarily formed by bulk degredation and have been defined as plastic materials less than 1000 nanometers (nm) [3]. There are secondarily derived through physical and mechanical breakdown, photodegradation, thermodegradation, and biodegradation of larger microplastics [4]. The size definition of nanomaterials is not isolated to plastics, but a symptom of a greater debate between scientists and regulators [5].

Nanomaterials traditionally describe particles that are intentionally produced at the nano-scale to take advantage of the physico-chemical properties available only at that size range [6]. Engineered or primary nanoplastics identified in personal care products, biomedical applications, and laboratory use are defined as less than 100 nanometers (nm) in a single dimension. For the purposes of this manuscript, we will define nanoplastics as particles that are less than 100 nm.

Unfortunately, due to their small size range, the quantity of nanoplastics in the environment currently cannot be measured. This is because the technologies to identify these small particles on a large scale have not yet been formulated. The traditional methodology of filtration cannot be used as the pores in most traditional containment centers are large enough to allow nanoplastics to pass through. Within the laboratory, nanotechnology techniques are in place to assess the small, known quantities to be characterized. These include dynamic light scattering, Raman spectroscopy, transmission electron microscopy, hyperspectral microscopy, and mass or size-based particle counters [7]. Further, laboratory assessments can modify nanoplastics to allow for their identification or quantification. This may be with the addition of a metallic core, or surface modifications including radioactive or fluorescent labelling [8–10]. Therefore, we await the analytic chemistry technologies. Further reading on the challenges of micro-, and subsequently nanoplastic, analyses are discussed here [11,12].

## 3. Particle characterizations and exposure

Nanoparticle potentials and toxicities are associated with the physico-chemical properties of the particles. This concept also holds true for nanoplastics. These particle characterizations include shape, size, chemical construct, and surface charge, each playing a key role in industrial and biocompatibility [13].

### 3.1. Shape, size, and surface area-to-mass ratios

Nanoplastics may be a variety of shapes. These include intentionally produced spheres for personal care products, angular particles generated from bulk fragmentation, or long and thin synthetic fibers. As mentioned above, nanomaterials are defined as having one dimension that measures less than 100 nm; therefore, nanoplastics can range greatly in size [3]. The size of the particle directly relates to nanoplastic surface area-to-mass ratios. The surface area-to-mass corresponds to the amount of surface are of an object (particle) within a given volume or collection of particles. For example: 100,10 nm sized particles can line up along the surface of a single 1 micron particle. Therefore, the large surface area-to-mass of the nanoparticles provide a greater surface for biological contact or chemical adsorption [1].

### 3.2. Chemical construct

Plastic polymers are generally formed using industrial chemicals to promote specific material characteristics (e.g., color, flexibility, hardness). According to the Society of the Plastics Industry (SPI) and as it pertains to plastic waste management guideline, there are 7 different types of plastics classified by their recycling code:(1)Polyethylene Terephthalate [PET(E)], (2) High-Density Polyethylene (HDPE), (3) Polyvinyl Chloride (PVC), (4) Low-Density Polyethylene (LDPE), (5) Polypropylene (PP), (6) Polystyrene, and (7) Other as not identified above, including Polycarbonate and polylactide (nylon) [14]. Each of these is made with differing general properties and commonly used in household products. While other modifications are available on the market due to material advances since the SPI guidelines were established (e.g., acrylics, acrylonitrile butadiene styrene, and polybrominated compounds), the toxicological assessments at the nanoscale have not been assessed. Exposure may not be limited to the baseline product or chemical modifications during degradation, but also chemical leaching of the additives may provide additional sources of contamination or toxicity [15].

### 3.3. Surface charge, functionalization, and chemical adsorption

Not only do nanoplastics have a polarization associated with their chemicals construct that may influence the hydrophilicity and hydrophobicity of the particle; but they can also adsorb chemical contaminants to their surface, transporting them within the environment or through a biological system [16–18]. Of the particles analyzed thus far, polyethylene has the greatest chemical sorption rates [19]. These chemical additions may act as a secondary toxicant or as a functionalized group on the surface of the particle, encouraging or discouraging biological interaction. These differential surface modifications and particle transformations will impact nanoplastic fate and toxicity [9,16,18,20].

### 3.4. Exposure

Given their size characteristics, nanoplastics easily escape traditional containment structures and solutions. Through disposal and degradation, nanoplastic particles can easily bypass landfill and wastewater containment, entering marine systems or becoming airborne; once in these forms, nanoplastics have the propensity for biological interactions associated with environmental and human exposure. As ongoing research continues to encourage the development of technologies and methodologies to aid in nanoplastic evaluation, it reveals the far-reaching scope of these particles.

## 4. Toxicity

Toxicology encompasses the biological relevance and adverse effects associated with exposure. As described above, nanoplastics have the propensity to be taken up by and enter animal and human systems. Studies are underway to establish the biological consequences associated with these exposures.

### 4.1. Bioaccumulation

Due to their small size, nanoplastics are widely distributed in the aquatic environment and can be easily ingested and taken up by a wide range of aquatic biota. Ingestion of microplastics represents an environmental concern for the health of the individual as well as for the trophic transfer of plastic contaminants to larger predators as in the case of transfer from algae, to zooplankton, and fish [21,22]. Small nanoplastics were found to directly absorb through the intestinal wall of mussels [20] and bioaccumulate in barnacles [23]. Evidence of plastic particles in the terrestrial environment confirm nanoplastic uptake by plants, earthworms, and in air pollution or aerosolized particulate matter [24].

As it pertains to biological activity, the nanoplastic chemical construct and surface charge influences cellular uptake rates in mussels and sea urchins [25,26]. Further, exposures to nanopolystyrene particles impair insulin and lipid peroxidation signaling cascades [27,28]. Interestingly, nanoplastic toxicity is differential as it relates to the health and anaerobic digestion activity of microbial communities [29]. Genotoxicity and modified genetic expression patterns has been identified after exposure in brine shrimp and zooplankton, leading to the hypothesis that nanoplastics may be mutagenic in high doses [28,30]. Co-incubation of polystyrene and polycarbonate nanoparticles promoted upregulation of stress responses within the innate immune system of fish [31]. The majority of work done in the field has been conducted in environmental models and this body of work has recently been reviewed [32].

### 4.2. Environmental outcomes and human health concerns

**Figure 1.**
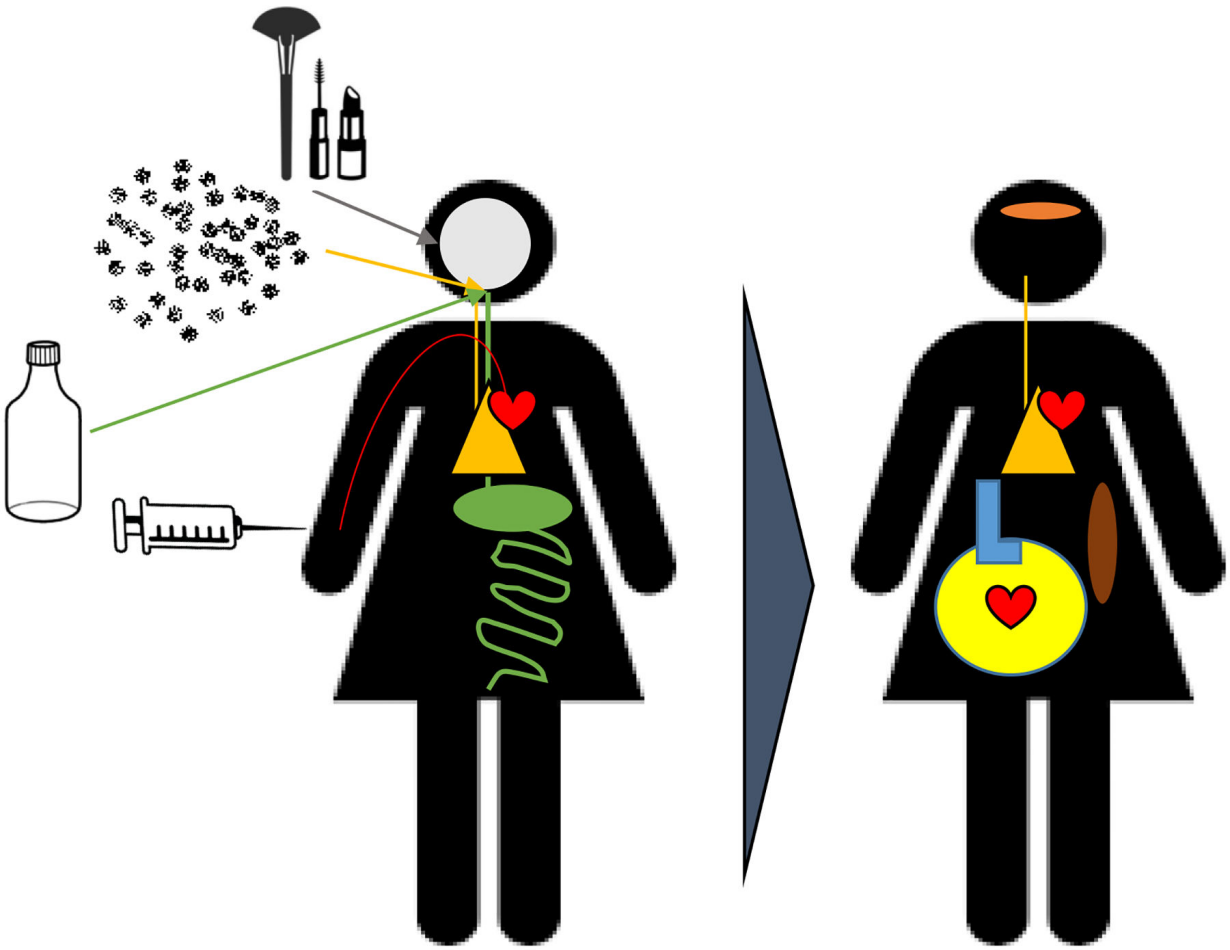
Diagram depicting the routes of nanoplastic exposure (i.e., ingestion, inhalation, dermal, and injection), potential primary systems of impact, and potential secondary toxicity associated with particle deposition.

With respect to human health, nanoplastic exposure may be through gastric ingestion, pulmonary inhalation, dermal application, and intentional injection (Figure 1). Exposure to nanoplastics may also be described as: (1) intentional means, as with the use of personal care products or biomedical applications [33], (2) unintentional exposure through intentional plastic use, as with consumption of bottled water [34], or (3) unintentional exposure, as with nanoplastic inhalation as a part of air pollution or digestion through food production [13,35–37]. Given the proliferation of nanoplastics within the food and water sources, gastric exposure is likely. However, as it pertains to the human environment, higher concentrations of airborne microplastics and extrapolated nanoplastics have been measured indoors [36].

While it is easiest or most comfortable to look at downstream contamination, separate from our homes and daily use activities, synthetic clothing is a primary source of airborne micro- and subsequent nanoplastics in the indoor and outdoor environments [35]. With regard to the widespread use of synthetic clothing and the amount of time that people spend indoors in domestic and occupational settings, this type and route of exposure need to be taken into consideration in future studies.

Recently, the potential human consumption of microplastics was assessed via meta-analysis [38]. Through these analyses, the authors calculated adults would be exposed to an average of 258 to 312 microplastic particles daily. The authors further determined that exposure would differential between the adults/children, sexes, and oral consumption or inhalation exposure [38]. Given the size disparity between micro- and nanoplastic particles, the estimate of nanoparticle exposure would be exponential.

#### 4.2.1. Gastric exposure

Current theories of human exposure and toxicity to nanoplastics identify ingestion as the primary exposure route [39]. While no direct toxicological assessments associated with the human ingestion of nanoplastics have been conducted, studies have identified that humans are consuming microplastics via their drinking water [34]. Further preliminary prospective analyses of human stool provide evidence of excretion of these particles, indicating exposure through food consumption [40]. When combined with studies of ingestion uptake in environmental models, it is evident that systemic up of nanoplastics in humans is likely. However, while concerns regarding human exposure via agroecosystems persist, analytical studies focused on known quantities of ingestion, paired with intestinal uptake, excretion, and particle fate have not been conducted [22].

#### 4.2.2. Pulmonary exposure

Second to nanoplastic ingestion is inhalation as a plausible route of human exposure. This may occur through indoor activities as identified above, or through the drying of contaminated waterways or wastewater [39]. Inhalation of nanosized particles or ultrafine air pollution (PM_0.1_) is associated with many health effects [41,42]. Particles within this size range deposit deep within the lung and remain in the alveolar space or translocate to other regions of the body [43–45]. As it pertains to plastics, through case study analyses [46], airborne microplastics exposures are known to cause disease (i.e., inflammation and cancer) after occupational exposure [36]. Further, animal studies suggest an increase in pulmonary inflammation associated with occupational exposures [47].

#### 4.2.3. Injection exposure

Studies have been conducted using nanoplastic injection as an exposure route. These studies primarily evaluate material translocation and excretion. Interestingly, using *ex vivo* assessment, our laboratory has determined that fluorescently labeled 20 nm polystyrene nanoparticles particles can cross the placental barrier and enter the fetal compartment via the umbilical vein within 90 minutes of infusion into the maternal uterine artery [48].

#### 4.2.4. Dermal exposure

Nanoplastics have been identified in personal care products, specifically facial scrubs [33], leading to the direct application of these materials onto the surface of the skin. While no studies to date have evaluated whether nanoplastics can cross the skin barrier, a single study evaluated engineered nanomaterials applied to textiles and identified that uptake of particles within this size range crossing intact skin is very low [49].

#### 4.2.5. In vitro studies

*In vitro* assessments investigate the local toxicities of particle-cellular interactions, making the assumption of systemic uptake, nanoplastic translocation, and deposition from the original site of exposure. The systemic outcomes associated with nanoparticle exposure are being elucidated; however, in each laboratory application of nanoplastic to the biological environment, toxicity has been identified.

Few cellular studies have been conducted to identify the cytotoxic effects of nanoplastic exposure and biological interactions. Co-incubation of nano-sized polystyrene and polyethylene particles have culminated in impaired the cellular metabolism of human lung cells [50] and increased oxidative stress [51] in epithelial and cerebral cell cultures. Further, the nanoplastic physoicochemical properties including size and surface modifications will directly affect cellular uptake and function in the forms of membrane disturbances, energy production, and oxidative stress [20,52].

#### 4.2.6. Particle translocation and secondary impacts

While many models consider the direct exposure of nanoplastics, future considerations must be made as it pertains to secondary toxicity associated with particle translocation and deposition (Figure 1). Often, the organs and systems considered as it pertains to nanomaterial transport and systemic toxicity are the vasculature, lymphatics, and filter organs (e.g. liver, kidney, spleen) [43]. Unfortunately, the majority of this work has yet to be conducted.

However, maternal-fetal models of exposure provide crucial data regarding translocation, deposition, and physiological barrier function. Within these assessments, nanoplastics have been identified within the embryonic tissues of zebrafish [53]. Recent evidence from our laboratory identifies the translocation of nano-sized polystyrene particle from the maternal to the fetal compartment, across the placental barrier within 70 minutes of injection into the uterine artery [48]. This perturbation of the placental barrier was echoed in a size-dependent manner wherein, nanopolystyrene particles were taken up by placental cells and translocated between fetal and maternal compartments in human placenta [54,55].

Taking into account particle translocation within the maternal system, recent preliminary evidence from our laboratory indicates the propensity of nanoplastic to migrate out of the maternal lungs within 24-hours after pulmonary exposure, depositing within the liver, spleen, and kidney [8]. Further, within our maternal-fetal model, we were able to detect 20 nm fluorescently-labelled polystyrene particles within the fetus, depositing within the placenta, heart, liver, and brain [8]. However, the local effects within fetal tissues or the lifelong outcome of this nanoplastic deposition is currently unknown.

## 5. Challenges and conclusions

At the present time, it is established that nanoplastic particles can cross biological membranes and influence cellular signaling; however, the cellular and systemic toxicities associated with these exposures have yet to be revealed. Future studies also must identify environmentally-relevant concentrations and take into account the nanoplastic physicochemical properties of each analyzed.

Plastics and their constituents are produced at a faster rate than their toxicities can be evaluated. For example, Bisphenol A (BPA) found industrial use in the mid-1950’s in the production of polycarbonate plastic and after initial assessments, was deemed safe for food packaging [56]. Low dose exposure to BPA was later identified as an endocrine disrupting compound with possible carcinogenic properties and subsequently banned for food product use in Canada, the EU, and the US between 2008 and 2012 [57]. In its place, BPA analogs Bisphenol S and F (BPS and BPF, respectively) are incorporated in consumer products to provide the same merchandise quality [58,59]. Unfortunately, given the novelty of these compounds, full toxicological assessments have not been completed and early results are conflicting [60–62]. In this example, the fields of engineering and chemistry have acted at a faster rate than the toxicological assessments of the new compounds can be properly conducted.

Further, with respect to the management of discarded plastics, the use of reverse polymerization is well documented. Concerning to toxicologists is the occupational and environmental exposures associated with reforming the chemical identity of these manipulated compounds and the intermediary gaseous components released during the process. However, there are few management strategies currently available to control plastic waste.

Understanding material fate and the toxicological effects of nanoplastics requires a collaborative effort from a wide variety of professionals including environmentalists, waste management specialists, chemists, engineers, and toxicologists. Recently, Rutgers University hosted a conference focused on the Impacts of Microplastics in the Urban Environment. At this meeting, the organizers had the foresight to invite experts in each of these fields to present their current work and encourage an open dialogue. Continued communication and engagement between these groups will allow collaborative efforts to identify a better understanding of particle properties, waste management strategies, changes to the properties over the plastic lifecycle, and the biological relevance of these differing properties.
